# Emergence of Multiplex Communities in Collaboration Networks

**DOI:** 10.1371/journal.pone.0147451

**Published:** 2016-01-27

**Authors:** Federico Battiston, Jacopo Iacovacci, Vincenzo Nicosia, Ginestra Bianconi, Vito Latora

**Affiliations:** 1 School of Mathematical Sciences, Queen Mary University of London, London E1 4NS, United Kingdom; 2 Dipartimento di Fisica ed Astronomia, Università di Catania and INFN, I-95123 Catania, Italy; Universiteit Gent, BELGIUM

## Abstract

Community structures in collaboration networks reflect the natural tendency of individuals to organize their work in groups in order to better achieve common goals. In most of the cases, individuals exploit their connections to introduce themselves to new areas of interests, giving rise to multifaceted collaborations which span different fields. In this paper, we analyse collaborations in science and among movie actors as multiplex networks, where the layers represent respectively research topics and movie genres, and we show that communities indeed coexist and overlap at the different layers of such systems. We then propose a model to grow multiplex networks based on two mechanisms of intra and inter-layer triadic closure which mimic the real processes by which collaborations evolve. We show that our model is able to explain the multiplex community structure observed empirically, and we infer the strength of the two underlying social mechanisms from real-world systems. Being also able to correctly reproduce the values of intra-layer and inter-layer assortativity correlations, the model contributes to a better understanding of the principles driving the evolution of social networks.

## Introduction

More often than not the agents of a social system prefer to combine their efforts in order to achieve results that would be otherwise unattainable by a single agent alone. A relevant role in the organisation of such systems is therefore played by the emerging patterns of collaboration within a group of individuals, which have been widely and thoroughly investigated in the last few decades [1, 2]. In a collaboration network, two individuals are considered to be linked if they are bound by some form of partnership. For instance, in the case of scientific collaborations, the nodes of the networks correspond to scientists and the relationship between two authors is testified by the fact that they have co-authored one or more papers [3]. Another well-known example of collaboration network is that of co-starring graphs, where the nodes represent actors and there is a link between two actors if they have appeared in the same movie.

The study of large collaboration systems has revealed the presence of a surprisingly high number of triangles in the corresponding networks [4, 5]. This indicates that two nodes with a common neighbour have a higher probability to be linked than two randomly chosen nodes. This effect, known as *transitivity* [1], can be easily explained in terms of a basic mechanism commonly referred to as *triadic closure* [6], according to which two individuals of a collaboration network have a high probability to connect after having been introduced to each other by a mutual acquaintance [4, 7, 8]. Some other works have pointed out that triadic closure can also explain other empirical features of real-world collaboration networks, including fat-tailed degree distributions and correlations between the degrees of neighbouring nodes [9, 10].

Another remarkable feature often observed in social and collaboration networks is the presence of meso-scale structures in the form of *communities*, i.e. groups of tightly connected nodes which are loosely linked to each other [11]. Interestingly, structural communities quite often correspond to functional groups [12].

An important observation is that not all the links of a collaboration network are equal, since collaborations can often be classified into a number of different categories. For instance, scientific co-authorship can be classified according to the research field, while actors often appear in movies of different genres. In these cases, a collaboration network is better described in terms of a *multi-layer* or *multiplex network* [13, 14] where links representing collaborations of a specific kind are embedded on a separate layer of the network, and each layer can have in general a different topology. Great attention has been recently devoted to the characterisation of the structure [15–20] and dynamics [21–24] of multi-layer networks. In particular, various models to grow multiplex networks have appeared in the literature, focusing on linear [25] or non-linear [26] preferential attachment, or on weighted networks [27]. Less attention has been devoted to define and extract communities in multiplex networks [28, 29], for instance by mean of stochastic block models [30, 31].

In this work we investigate the multiplex nature of communities in collaboration networks and we propose a simple model to explain the appearance, coexistence and co-evolution of communities at the different layers of a multiplex. Our hypothesis is that the formation of communities in collaboration networks is an intrinsically multiplex process, which is the result of the interplay between intra-layer and inter-layer triadic closure. For instance, in the case of scientific collaborations, multiplex communities naturally arise from the fact that scientists may collaborate with other researchers in their principal field of investigation and with colleagues coming from other scientific disciplines. Analogously, actors can prefer either to specialise in a specific genre or instead to explore different (sometimes dissonant) genres, and these two opposite behaviours undoubtedly have an impact on the kind of meso-scale structures observed on each of the layers of of the system. The generative model we propose here mimics two of the most basic processes that drive the evolution of collaborations in the real world, namely intra- and inter-layer triadic closure, and is able to explain the appearance of overlapping modular organisations in multi-layer systems. We will show that the model is able to reproduce the salient micro-, meso- and macro-scale structure of different real-world collaboration networks, including the multi-layer network of co-authorship in journals of the American Physical Society (APS) and the multiplex co-starring graph obtained from the Internet Movie Database (IMDb).

## Results

### Empirical analysis

We start by analysing the structure of two multiplex collaboration networks from the real world. The first multiplex is constructed from the APS co-authorship data set, and consists of four layers representing four sub-fields of physics (respectively, Nuclear physics, Particle physics, Condensed Matter I, and Interdisciplinary physics). In particular, we considered only scientists with at least one publication in each of the four sub-fields, and we connected two scientists at a certain layer if they had co-authored at least a paper in the corresponding sub-field. The second multiplex is constructed from the Internet Movie Database (IMDb) and consist of four layers respectively representing the co-starring networks of actors with at least one participation in four different genres, namely Action, Crime, Romance, and Thriller movies. The basic structural properties of each layer of the two multiplexes are summarised in Table 1 (see Methods for more information about the data sets).

**Table 1:** Basic properties of real-world multiplex collaboration networks. We report the number of nodes *N*, the average degree 〈*k*〉 and the clustering coefficient *C* for each layer of a subset of the APS and IMDb data sets. In particular, we focus on the multiplex collaboration network of all scientists active in Nuclear, Particle, Condensed Matter I and Interdisciplinary physics, and the multiplex collaboration network of all actors starring in Action, Crime, Romance and Thriller movies. All the layers of APS have a clustering coefficient *C* in the range [0.24, 0.30]. Conversely, the values of *C* of all the IMDb layers are in the range [0.56, 0.61].

**APS**	*N*	〈*k*〉	*C*
Nuclear (N)	1238	4.75	0.27
Particle (P)	1238	4.66	0.30
Cond. Matt. I (CM)	1238	10.29	0.24
Interdisciplinary (I)	1238	7.37	0.26
**IMDb**	*N*	〈*k*〉	*C*
Action (A)	55797	83.56	0.61
Crime (C)	55797	82.30	0.58
Romance (R)	55797	86.00	0.59
Thriller (T)	55797	77.75	0.56

Since we are interested in assessing the role of intra- and inter-layer triadic closure in the formation of meso-scale multiplex structures, we quantified the transitivity of each layer through the clustering coefficient *C* [4], which takes values in the interval [0, 1] (see Methods). We notice that the four layers of each data set have similar values of clustering, ranging respectively in [0.24, 0.3] in the case of APS and in [0.56, 0.61] for IMDb. As we will discuss in the following, by focusing on layers having comparable clustering we will be able to perform a comparison between the structure of these real-world multiplex networks and the proposed model in its simplest formulation.

**Fig 1:**
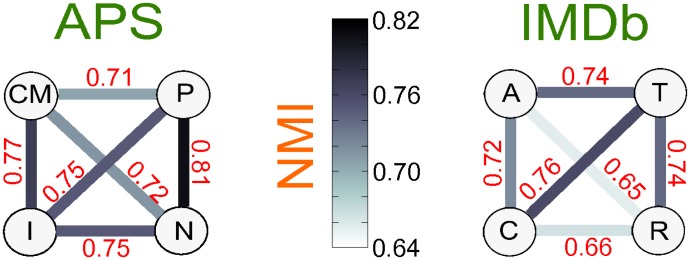
Similarity of communities at the different layers of real-world collaboration networks. In each of the two graphs nodes represent the layers of the multiplex (APS on the left and IMDb on the right) and the edges are coloured according to the value of the normalised mutual information for the community decompositions at the corresponding pairs of layers.

The multiplex nature of communities in collaboration networks can be measured by means of the normalised mutual information (NMI) [32] (see Methods), which quantifies the similarity between the partition in communities observed in two different layers of a multiplex. The normalised mutual information takes values in [0, 1]. In general, higher values of NMI correspond to more similar partitions. The values of NMI for each pair of layers in APS and IMDb are shown in Fig 1. It is interesting to notice that in general pairs of layers corresponding to related subjects or genres exhibit higher values of NMI. This is for instance the case of Nuclear Physics and Particle Physics in APS. Similarly, in the IMDb network we observe a higher similarity between the communities at the three layers representing respectively Thriller, Crime and Action genres. Conversely, the layer of Romance movies displays a different modular structure from Crime and Action. Notice also that the level of similarity between the communities of two layers can vary substantially, despite the four layers of each multiplex have roughly the same clustering coefficient.

### Model

In the following Section we introduce a model to grow collaboration networks with tunable multiplex community structure, able to reproduce the patterns observed in the considered real-world systems. Let us consider for simplicity the case of a multiplex with *M* = 2 layers, and assume that initially each layer consists of a clique of *n*_0_ nodes. Then at each time step *t* a new node is added to the network, with *m*^[1]^ edge stubs to be connected on layer 1 and *m*^[2]^ other stubs to be connected on layer 2. The multiplex network grows according to the following rules:

- *Layer selection*. The newly arrived node *i* selects one of the two layers {1, 2} uniformly at random. Let us label the first selected layer with the index *a*. The first edge of *i* is connected to one of the existing nodes on that layer, chosen uniformly at random, that we call *n*_*a*_.
- *Intra-layer triadic closure (I)*. The remaining *m*^[*a*]^-1 edges of node *i* on layer *a* are attached with probability *p*^[*a*]^ to one of the first neighbours of *n*_*a*_, chosen uniformly at random, and with probability 1 − *p*^[*a*]^ to one of the nodes of layer *a*, chosen uniformly at random.
- *Inter-layer triadic closure*. When all its *m*^[*a*]^ edges on layer *a* have been created, node *i* starts connecting on the other layer *b* with *m*^[*b*]^ edges. The first link in layer *b* is created with probability p* to the same node *n*_*a*_, and with probability 1 − p* to one of the other nodes, chosen uniformly at random. The node to which this first link is attached is called *n*_*b*_.
- *Intra-layer triadic closure (II)*. The remaining *m*^[*b*]^-1 links at layer *b* are attached with probability *p*^[*b*]^ to one of the first neighbours of *n*_*b*_ chosen uniformly at random, and with probability 1 − *p*^[*b*]^ to one of the nodes at layer *b*, chosen uniformly at random.

**Fig 2:**
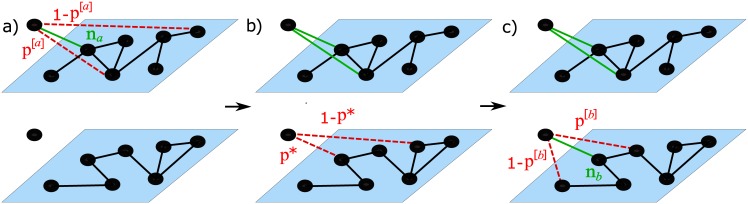
Schematic representation of network growth with intra-layer and inter-layer triadic closure. A newly arrived node *i* creates *m*^[1]^ new edges on layer 1 and *m*^[2]^ new edges on layer 2. The new node starts by choosing at random one of the two layers {1, 2}. We indicate the first chosen layer using the label *a*. a) The first link of the new node is connected to one of the nodes of layer *a*, chosen uniformly at random and called *n*_*a*_ (solid green line). Each of the remaining *m*^[*a*]^ − 1 links is attached with probability *p*^[*a*]^ to a neighbour of the previously chosen node (intra-layer triadic closure) or with probability 1 − *p*^[*a*]^ to one of the nodes at layer *a*, chosen uniformly at random (dashed red lines). b) Afterwards, the new node starts connecting on the other layer *b*. The first link on layer *b* is created to node *n*_*a*_ with probability p*, or to one of the other nodes at layer at random with probability 1 − p*. We call *n*_*b*_ the first node to which *i* attaches on layer *b*. c) Each of the *m*^[*b*]^ − 1 remaining edges on layer *b* are attached with probability *p*^[*b*]^ to one of the neighbours of *n*_*b*_, and with probability 1 − *p*^[*b*]^ to one of the nodes on layer *b*, chosen uniformly at random.

This general model has five parameters to be tuned, namely the number of new edges *m*^[1]^ and *m*^[2]^ brought by each new node on each of the two layers, which determine the average degree on each layer, and the three probabilities *p*^[1]^, *p*^[2]^, and p*, which are respectively responsible for the formation of intra- and inter-layer triangles. In fact, by varying the parameters *p*^[1]^ and *p*^[2]^ we can tune the strength of the intra-layer triadic closure mechanism, i.e the probability to form triangles on each of the two layers. In particular, larger values of *p*^[1]^ and *p*^[2]^ will foster the creation of a larger number of triangles in layer 1 and layer 2 respectively. Conversely, the parameter p* tunes the inter-layer triadic closure mechanism, and in particular high values of p* correspond to a higher probability that the neighbourhoods of node *i* at the two layers will exhibit a certain level of overlap. These two simple attachment rules, namely intra-layer and inter-layer triadic closure, aim to describe the real mechanisms characterising the evolution of collaboration networks. We argue that, for instance, scientists do not tend to collaborate with other scientists at random. Instead, they usually exploit the neighbourhoods of their collaborators in a specific field (*intra-layer* triadic closure). Similarly, when opening themselves to new scientific fields, a researcher usually takes into account the neighbourhoods of their past colleagues from previous collaborations in other fields (*inter-layer* triadic closure). A schematic representation of the model is depicted in Fig 2.

It has been recently shown [10] that in a single-layer network scenario the interplay between random attachment and triadic closure leads to a network growth in which the attachment probability (i.e., the probability for an existing node to receive one of the new edges) is a sub-linear function of the degree, and produces networks with non-trivial community structure, as long as the link density is not too high. In the multi-layer model we propose, the further addition of an inter-layer triadic closure mechanism allows to tune at will the overlap between the community structures at the different layers.

## Validation in a Simple Scenario

To assess the ability of the model to reproduce the organisation of communities in multiplex networks, we start by considering a simple scenario, i.e. the case in which the layers of the multiplex have the same density (*m*^[1]^ = *m*^[2]^ = *m*) and the same clustering coefficient (*p*^[1]^ = *p*^[2]^ = *p*). We show that this simplified version of the model is already able to reproduce both the different levels of similarity between community structures at different layers, and the microscopic patterns of intra-layer and inter-layer degree correlations observed in the real-world collaboration multiplexes of APS and IMDb.

**Fig 3:**
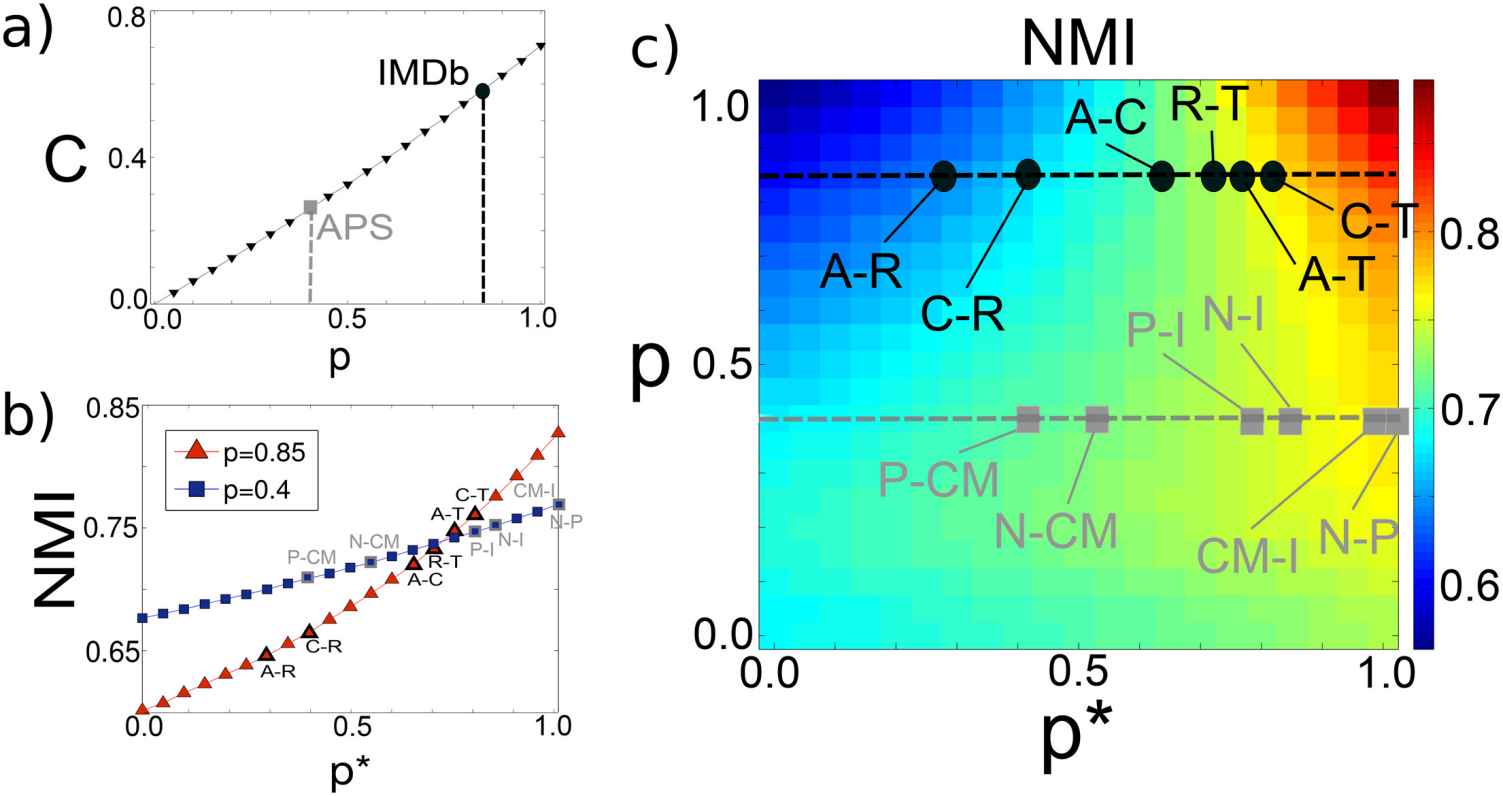
Model calibration in a simple scenario. We show the values of *p* and p* extracted for the different pairs of layers of the four-layer collaboration networks of APS and IMDb. (a) The clustering coefficient *C* depends exclusively on the parameter *p*, which tunes intra-layer triadic closure. Since all the layers of those two multiplex networks have comparable clustering coefficients, we are able to determine the value of the parameter *p* in each of the two cases. (b) For each pair of layers, we can also determine the value of the inter-layer triadic closure parameter p* by setting it equal to the value which yields an organisation in communities characterised by a value of NMI compatible with that observed in the real network.

In Fig 3(a), we report the values of the clustering coefficient *C* (which, by construction, does not depend on the parameter p*) for several realisations of the model (see Methods). As expected, the clustering coefficient of each layer is a linearly increasing function of the parameter *p*, which tunes the strength of intra-layer triadic closure. This means that, if we consider a real-world multiplex network whose layers have approximately the same value of clustering coefficient *C*, we can set the value of the parameter *p* of the model accordingly. This is for instance the case of the four-layer multiplex networks of APS and IMDb constructed in the previous Section, where all the layers have comparable levels of clustering. We obtain *p* = 0.40 for APS and *p* = 0.85 for IMDb, respectively.

In Fig 3(c) we show, as a colour-map, the values of NMI of the networks obtained through the proposed model by using different combinations of the parameters *p* and p* (see Methods). It is evident that, in spite of its simplicity, the model can yield a quite rich variety of multiplex networks. In agreement with intuition, when both *p* and p* are large one obtains multiplexes with higher values of NMI. In fact, in this regime both the intra-layer and inter-layer triadic closure mechanisms are strongly affecting the network evolution and, as a consequence, it is likely that the new node joining the network will close a triad on both layers in the same region of the network. As a consequence, each layer will have a strong community structure (large *p*) which is pretty much correlated to the one present on the other layer, due to the large value of inter-layer triadic closure p*. Conversely, if the inter-layer parameter p* is small we will obtain layers whose partitions in communities are poorly correlated when *p* is large (blue region in the phase space of Fig 3, while the NMI is only marginally larger when *p* is very small (bottom-left corner of the phase space).

**Fig 4:**
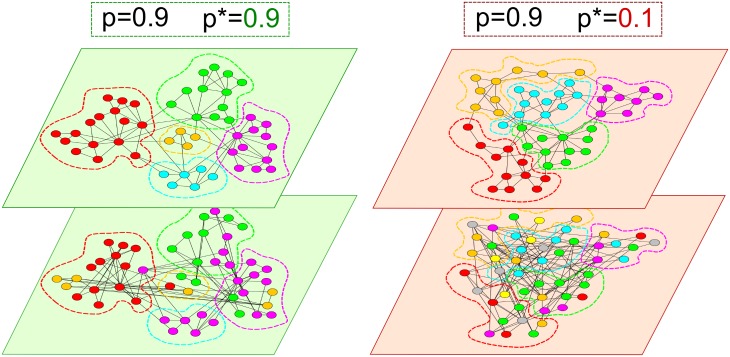
Layers with similar or dissimilar community structures. We show the effect of the value of the inter-layer triadic closure parameter p* on the multiplex community structure. The two top layers show two typical realisations of the simplest version of the network model with *N* = 50, *m*^[1]^ = *m*^[2]^ = 2 and *p*^[1]^ = *p*^[2]^ = 0.9. Nodes belonging to the same community are given the same colour and are drawn close to each other. The two layers at the bottom of each multiplex are obtained by setting, respectively, p* = 0.9 (left) and p* = 0.1 (right). The nodes maintain the same placement in space on the second layer, but are coloured according to the community they belong in that layer (colours are chosen in order to maximise the number of nodes that have the same colour in the two layers). It is evident that the community structures of the two layers on the left, corresponding to p* = 0.9, are very similar, while the partition into communities of the upper layer on the left panel is substantially different from the one observed in the bottom layer of that multiplex.

In Fig 4 we report two realisations of the multiplex network model with *N* = 50, *m*^[1]^ = *m*^[2]^ = 2 and *p*^[1]^ = *p*^[2]^ = 0.9, respectively for p* = 0.9 (left) and p* = 0.1 (right). Nodes belonging to the same community are reported using the same colour, and the colour chosen for each community in the second layer (bottom) corresponds to the colour of the community in the first layer (top) for which the node overlap between the communities is maximum. These two examples help explain the role of the parameter p* in shaping the inter-layer modular structure of the network. For p* = 0.9 (left panel) the community structures of the two layers are closely matched (this situation corresponds to the high values of NMI found in the top-right region of the heat-map in Fig 3), while for p* = 0.1 (right panel) the communities at the two layers are uncorrelated (low values of NMI in the top-left of the heat-map in Fig 3).

Differently from the clustering coefficient *C*, the values of the normalised mutual information NMI depend on both *p* and p*. Having already determined a candidate value of *p* for each multiplex by fitting the clustering coefficient of its layers, we can determine the strength of the inter-layer triadic closure mechanism by fitting the NMI. Remarkably, for any fixed value of *p*, the simplest formulation of our model is able to reproduce all the values of NMI observed in the real-world networks by just tuning the parameter p*, with the exception of the pair Nuclear-Particle physics which is slightly out of the plane with an NMI value of 0.81 (represented on the right border of the plane which corresponds to NMI = 0.79). We would like to note here that the model is able to produce a remarkably wide range of values of NMI, which span the whole interval [0.6, 0.9].

We further validate the model by showing that, using the inferred parameters (*p*,p*), we are able to reproduce quite well the patterns of degree-degree correlations observed in the real-world collaboration multiplexes.

**Fig 5:**
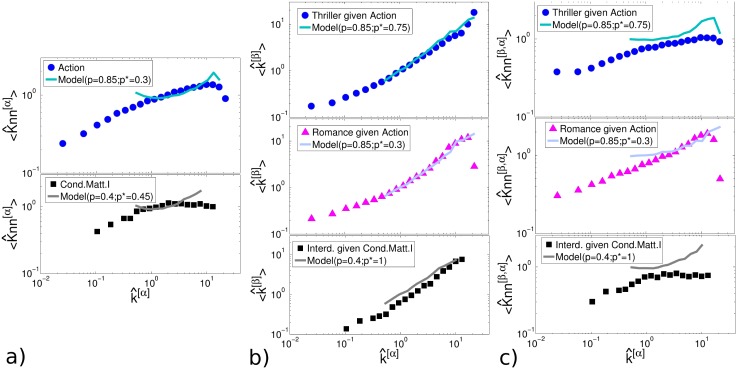
Intra-layer, inter-layer and mixed assortativity in collaboration networks. We show the intra-layer (a), inter-layer (b) and mixed (c) degree-correlations for couples of layers of the IMDb and APS collaboration networks. Real data (dots) are compared with the results of our model (solid lines) generated with the extracted values *p* and p*. The symbols (^) indicate that the reported quantities (both for the model and the data) have been normalised to the values observed in the corresponding configuration model. As shown, the model is in general able to correctly capture the assortative trends of the three different types of correlations. Very good agreement with the data is attained in the case of the movie actor collaboration network. Less precise results are obtained for the APS network, where we deal with a system of considerably smaller size.

Indeed, for each pair of layers *α* and *β* we analysed:

1. the intra-layer degree correlations, by looking at the average degree 〈*Knn*^[*α*]^〉 of the first neighbours on layer *α* of nodes having a certain degree *k*^[*α*]^, as a function of *k*^[*α*]^;
2. the inter-layer degree correlations, by looking at the average node degree 〈*k*^[*β*]^〉 on *β* given the degree *k*^[*α*]^ on *α*;
3. the mixed degree correlations by looking at the average mixed node degree 〈*Knn*^[*β*,*α*]^〉 given the degree *k*^[*α*]^ on *α*;

(see Methods for details). The results are shown in Fig 5 for some significant examples. Dots represent the values measured on the real-world networks, while solid lines correspond to the values obtained in the corresponding multiplex models. Symbols with a hat (^) indicate that the value of the considered variables, for both the model and the data, have been normalised to the values of the corresponding configuration model to allow a comparison (see Methods). It is interesting to notice that the model reproduces quite well the three types of degree correlations in the IMDb multiplex, both in the case of high *p* and high p* (Action, Thriller given Action) and the case of small *p* and small p* (Action, Romance given Action). A quantitative comparison of the the power-law fits of the curves is reported in Table 2. As an example from APS we consider Condensed Matter I and Interdisciplinary physics (small *p* and high p*). In this case we observe marked differences in the correlations measured in the real-world network and in the model network, for both 〈*Knn*^[*α*]^〉 and 〈*Knn*^[*β*,*α*]^〉. In particular, the model seems to overestimate degree correlations. These discrepancies are probably due to the relatively small number of nodes (only 1238) in the considered data subset.

**Table 2:** Quantitative comparison between the curves obtained from the model and the data for the inter-layer degree correlations the and mixed degree correlations. The curves have been fitted using a function of the form *f*(*x*) ∼ *x*^*γ*^; the *γ* parameter is reported for the corresponding curves in Fig 5.

Layers’ pair	〈*k*^[*β*]^〉		〈*Knn*^[*β*,*α*]^〉	
	*γ*_data_	*γ*_model_	*γ*_data_	*γ*_model_
Interd. given Cond. Matt. I	0.98	0.85	0.14	0.30
Thriller given Action	0.83	0.84	0.16	0.17
Romance given Action	0.89	0.87	0.30	0.27

Although our intention was not to exactly reproduce all the features observed in real-world collaboration multiplex networks, it is interesting to observe that the two mechanisms of inter-layer and intra-layer triadic closure play an important role in determining the degree-degree correlations in such networks. We also notice that the degree distributions of the layers in the synthetic networks are compatible with the stretched exponential functional forms introduced and discussed in Ref. [10].

## Model Calibration for Generic Multiplex Networks

We now discuss how to calibrate the model in the most general case in which the layers might possibly have different edge density, i.e. *m*^[1]^ ≠ *m*^[2]^, and different clustering, i.e. *p*^[1]^ ≠ *p*^[2]^. As an example, we consider the co-authorship networks of the same four sub-fields of physics (namely, Nuclear, Particle, Condensed Matter I and Interdisciplinary physics) used to construct the four-layer APS multiplex (cf. Table 1 and Fig 1). However, we focus here on all two-layer multiplex networks obtained by combining two networks at a time, so that, for instance, a node appears in the Nuclear-Particle (N-P) multiplex network if the corresponding author has published papers in both sub-fields. In general, the obtained multiplex networks are composed by layers with different edge density and different clustering coefficients, as shown in Table 3, thus we need to set separately the four parameters of the model *p*^[1]^, *p*^[2]^, *m*^[1]^ and *m*^[2]^.

**Table 3:** Basic properties of duplex networks in APS. We consider all the possible multiplex networks with *M* = 2 layers obtained from combinations of the APS collaboration networks corresponding to the four sub-fields Nuclear, Particle, Condensed Matter I and Interdisciplinary Physics. For each duplex, we report the number of nodes *N*, the average degree on the two layers 〈*k*^[1]^〉 and 〈*k*^[2]^〉, and the values of the clustering coefficients *C*^[1]^ and *C*^[2]^.

**Layer 1**	**Layer 2**	*N*	〈*k*^[1]^〉	〈*k*^[2]^〉	*C*^[1]^	*C*^[2]^	*NMI*
Nuclear	Particle	6572	6.88	7.46	0.56	0.56	0.83
Nuclear	Cond. Matt. I	3828	4.53	7.20	0.43	0.34	0.71
Nuclear	Interdisciplinary	2556	4.15	5.39	0.37	0.33	0.72
Particle	Cond. Matt. I	3774	5.70	7.82	0.53	0.40	0.71
Particle	Interdisciplinary	2502	4.82	5.66	0.49	0.39	0.74
Cond. Matt. I	Interdisciplinary	27257	10.34	7.05	0.55	0.64	0.82

**Fig 6:**
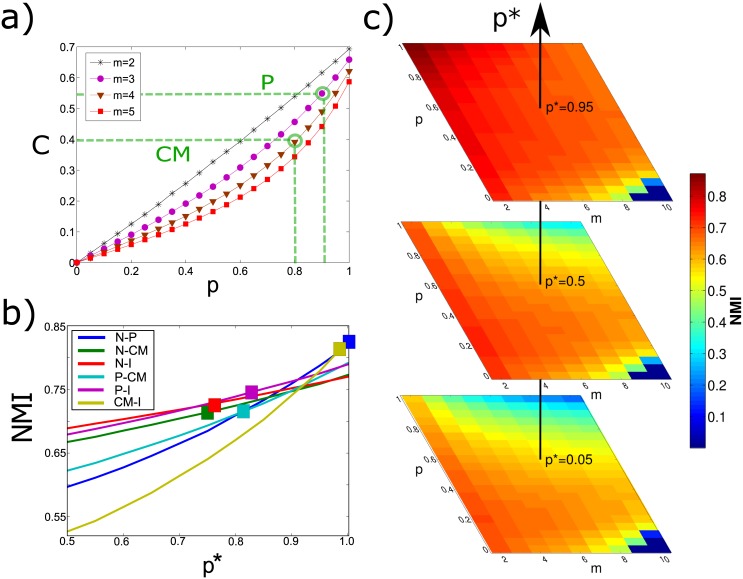
Model calibration. In panel a) we show the dependence of the clustering coefficient *C* on the intra-layer triadic closure parameter *p* for different values of the parameter *m*, which sets the layer’s average degree. In the multiplex consisting of the layers Particle (P) and Condensed Matter I (CM), the average degree of each layer corresponds, respectively, to *m*^[1]^ = 3 and *m*^[2]^ = 4. The value of *p*^[1]^ and *p*^[2]^ are determined to match the clustering coefficients *C*^[1]^ and *C*^[2]^. In panel b), after having determined *m*^[1]^, *m*^[2]^, *p*^[1]^ and *p*^[2]^ for all the pairs of layers in the APS dataset, we run the model with such parameters for different value of p* and infer, for each pair, the value of the inter-layer triadic closure parameter p* yielding a value of NMI compatible with that observed (see Table 1 for layers’ acronyms). In panel c) we plot a heat-map of the NMI as a function of *p* and *m*, respectively for low (0.05), intermediate (0.50) and high (0.95) values of p* in the model with *m*^[1]^ = *m*^[2]^ = *m* and *p*^[1]^ = *p*^[2]^ = *p*. An increase in the link density of the layers produces a less correlated community structure in the two layers, even if the inter- and intra-layer triadic closure strengths are high.

We start by observing that the average degree of a synthetic layer is 〈*k*〉 ≃ 2*m*, where *m* is the number of edge stubs connected by a newly arrived node, so that the parameters *m*^[1]^, *m*^[2]^ of the model can be set respectively equal to $\left[\frac{\langle k^{\left[1\right]}\rangle }{2}\right]$ and $\left[\frac{\langle k^{\left[2\right]}\rangle }{2}\right]$, where 〈*k*^[1]^〉 and 〈*k*^[2]^〉 are the measured average degrees of the two layers (numbers are approximated to the closest integers). Similarly, as we show in Fig 6(a), the clustering coefficient *C*^[*α*]^ of a layer *α* is univocally determined by *p*^[*α*]^, as soon as *m*^[*α*]^ is fixed. In Fig 6(a) we show how the values of *C*^[*α*]^ change as a function of *p*^[*α*]^, for different values of *m*^[*α*]^. Hence, the values of the intra-layer triadic closure parameters *p*^[1]^ and *p*^[2]^ can be set in order to match the values of clustering coefficient observed in each of the two layers. The only parameter yet to be determined is p*. However, if we set the values of *m*^[1]^, *m*^[2]^, *p*^[1]^, and *p*^[2]^ to match the densities and clustering coefficients of the layers, we can then run the model for different values of p* and look for the one which yields a value of NMI as close as possible to the one observed in the real two-layer multiplex. This procedure is sketched in Fig 6(b) for the six two-layer multiplexes in APS.

In order to better understand the role of the different parameters, in Fig 6(c) we report the values of NMI obtained from different realisations of the model with *m*^[1]^ = *m*^[2]^ = *m* and *p*^[1]^ = *p*^[2]^ = *p* for *m* varying in [2, 3, …, 10], and *p* varying in [0, 0.1, …, 1] at different values of p*, [0.05, 0.5, 0.95], corresponding respectively to low, intermediate and high inter-layer triadic closure strength. We see that the effect of the increase in the link density *m* of the layers leads to a decrease in the similarity of their community structures even for high values of *p* and p*.

It is interesting to notice that, although the generic version of the model depends on five parameters, respectively accounting for layer density (*m*^[1]^ and *m*^[2]^), triadic closure (*p*^[1]^ and *p*^[2]^), and inter-layer overlap of communities (p*), the values of those parameters can be easily set by measuring just the average degree and the average clustering coefficient of each layer, and the normalised mutual information between the community structures at the two layers. Again, the good agreement between the synthetic networks and the real-world datasets extends also to other structural properties, such as intra-layer and inter-layer degree correlations, which were thought to have little or no direct relation at all with triadic closure. These results suggest that triadic closure plays an unexpectedly central role in determining the structural properties of real-world multiplex collaboration networks.

## Discussion

Human collaboration patterns are inherently multifaceted and often consist of different interaction layers. Scientific collaboration is probably the most emblematic example. As a Ph.D. student you usually join the scientific collaboration network by publishing the first paper with your supervisor in a specific field. Afterwards, you start being introduced by your supervisor to other researchers in the same field, e.g. to some of his/her past collaborators, and you might end up working with them, creating new triangles in the collaboration network of your field (what we called intra-layer triadic closure). But it is also quite probable that some of your past collaborators will in turn introduce you to researchers working in another -possibly related- area (what we called an inter-layer triadic closure), so that you will easily find yourself participating in more than just one field, and the collaboration network around you will become multi-dimensional. Such multi-level collaboration patterns appear not to be specific of scientific production only, but are instead found in many aspects of human activity.

The multi-layer network framework provides a natural way of modelling and characterising multidimensional collaboration patterns in a comprehensive manner. In particular, we have argued that one of the classical mechanisms responsible for the creation of triangles of acquaintances, i.e. triadic closure, is indeed general enough to give also account for another interesting aspect of multi-level collaboration networks, namely the formation of cohesive communities spanning more than a single layer of interaction. It is quite intriguing that the simple model we proposed in this work, based just on the interplay between intra- and inter-layer triadic closure, is actually able to explain much of the complexity observed in the micro- meso- and macroscopic structure of multidimensional collaboration networks of different fields (science and movies), including not just transitivity but also intra- and inter-layer degree correlation patterns and the correspondence between the community partitions at difference layers. We also remark that such levels of accuracy in reproducing the features of real-world systems have been obtained without the introduction of ad-hoc ingredients.

The results reported in this paper suggest that, despite the apparent differences in the overall dynamics driving scientific cooperation and movie co-starring, triadic closure is a quite generic mechanism and might indeed be one of the fundamental processes shaping the structure of multi-layer collaboration systems. These findings fill a gap in the literature about modelling growing multidimensional networks, and pave the way to the exploration of other simple models which can help underpinning the driving mechanisms responsible for the emergence of complex multi-dimensional structures.

## Methods

### Data sets

We considered data from the APS and the IMDb collaboration networks. The APS collaboration data set is available from the APS website http://journals.aps.org/datasets in the form of XML files containing detailed information about all the papers published by all APS journals. The download is free of charge and restricted to research purposes, and APS does not grant to the recipients the permission to redistribute the data to third parties. We parsed the original XML files to retrieve, for each paper, the list of authors and the list of PACS codes. The PACS scheme provides a taxonomy of subjects in physics, and is widely used by several journals to identify the sub-field, category and subject of papers. We used the highest level of PACS codes to identify the ten main sub-fields of physics, and we considered only the papers published in Nuclear physics, Particle physics, Condensed Matter I and Interdisciplinary physics, respectively associated to high-level PACS codes starting with 1 (Particle physics), 2 (Nuclear physics), 6 (Condensed Matter I) and 8 (Interdisciplinary physics). We focused only on the authors who had at least one publication in each of the four sub-fields [19]. The co-authorship network of each of those four sub-fields constitutes one of the four layers of the APS multiplex. In particular, two authors are connected on a certain layer only if they have co-authored at least one paper in the corresponding sub-field. In the construction of the collaboration network of each sub-field we purposely left out papers with more than ten authors, which represent big collaborations whose driving dynamics might be more complex than just triadic closure.

The IMDb data set is made available at the website ftp://ftp.fu-berlin.de/pub/misc/movies/database/ for personal use and research purposes. The data set comes in the form of several compressed text files, and we used those containing information about actors, actresses, movies and genres. We focused only on the co-starring networks of four movie genres, namely Action, Crime, Romance, and Thriller [19], obtained by merging information about participation of actors and actresses to each movie. In particular, two actors are connected by a link on a given layer (genre) only if they have co-starred in at least one movie of that genre. We considered only the actors who had acted in at least one movie of each of the four genres. We chose to restrict our analysis to just four layers for both the APS and the IMDb data set, which allowed us to consider the simplest formulation of our model, in which all the layers have the same clustering coefficient *C*. The use of the APS and the IMDb data sets does not require any ethical approval.

### Transitivity and community structure

We measured the transitivity of each level by mean of the clustering coefficient *C* = (1/*N*)∑_*i*_ *C*_*i*_ [4], where *C*_*i*_:

$$C_{i}=\frac{\sum_{j\neq i,m\neq i}a_{ij}a_{jm}a_{mi}}{\sum_{j\neq i,m\neq i}a_{ij}a_{mi}}=\frac{\sum_{j\neq i,m\neq i}a_{ij}a_{jm}a_{mi}}{k_{i}\left(k_{i}-1\right)}.$$ (1)

The similarity of two community partitions can be measured through the normalised mutual information (NMI) [32]. In particular, given the two partitions $P_{\alpha }$ and $P_{\beta }$ respectively associated to layer *α* and layer *β*, we denote the normalised mutual information (NMI) between them as

$$NMI\left(P_{\alpha },P_{\beta }\right)=\frac{-2\sum_{m=1}^{M_{\alpha }}\sum_{m^{\prime }=1}^{M_{\beta }}N_{mm^{\prime }}\log\left(\frac{N_{mm^{\prime }}N}{N_{m}N_{m^{\prime }}}\right)}{\sum_{m=1}^{M_{\alpha }}N_{m}\log\left(\frac{N_{m}}{N}\right)+\sum_{m^{\prime }=1}^{M_{\beta }}N_{m^{\prime }}\log\left(\frac{N_{m^{\prime }}}{N}\right)}$$ (2)

where *N*_*mm*′_ is the number of nodes in common between module *m* of partition $P_{\alpha }$ and module *m*′ of partition $P_{\beta }$, while *N*_*m*_ and *N*_*m*′_ are respectively the number nodes in module *m* and in module *m*′. The partition in communities on each layer has been obtained through the algorithm Infomap [33].

### Synthetic multiplex networks

We created synthetic networks according to our multi-layer network model by starting, on each layer, from a seed graph consisting of a triangle of nodes and simulating the intra- and inter-layer triadic closure mechanism for *N* = 20000 nodes, for different values of the parameters *p* and p*. For each pair of values (*p*, p*) we computed the mean clustering coefficient *C* on each single layer and the normalised mutual information NMI of the community partitions of the two layers over 30 different realisations. As observed from simulations, once the parameters (*p*, p*) are fixed, the values of NMI and *C* do not vary substantially as the order *N* of the network increases. Notice that since the most simple formulation of the model we have set an identical value of *p* on both layers, the two layers will end up having the same clustering coefficient (up to small finite-size fluctuation).

### Degree correlations

We study the assortativity of real multiplex collaboration networks in terms of intra-layer, inter-layer and mixed degree correlations. The trend for intra-layer correlations is analysed by mean of the function $\langle K_{nn}^{\left[\alpha \right]}\left(k^{\left[\alpha \right]}\right)\rangle$, that is the average degree of the nearest neighbours on layer *α* of a node with given degree *k*^[*α*]^ on that layer. In particular, $\langle K_{nn}^{\left[\alpha \right]}\rangle$ is obtained as an average of $K_{nn,i}^{\left[\alpha \right]}$ over all nodes with the same degree *k*^[*α*]^. The node term can be computed as $K_{nn,i}^{\left[\alpha \right]}=\frac{\sum_{j\neq i}a_{ij}^{\left[\alpha \right]}k_{j}^{\left[\alpha \right]}}{k_{i}^{\left[\alpha \right]}}$, where $a_{ij}^{\left[\alpha \right]}$ are the entries of the adjacency matrix at layer *α*. Since such measure considers only a layer at a time, the layer index here is not strictly necessary but will be kept for symmetry with the other coefficients. It is interesting to notice that, in absence of intra-layer degree correlations, $\langle K_{nn}^{\left[\alpha \right]}\left(k^{\left[\alpha \right]}\right)\rangle$ is a constant, while $\langle K_{nn}^{\left[\alpha \right]}\left(k^{\left[\alpha \right]}\right)\rangle$ is an increasing (resp., decreasing) function of *k*^[*α*]^ if assortative (resp., disassortative) degree correlations are present.

To quantify inter-layer degree correlations we considered the quantity 〈*k*^[*β*]^(*k*^[*α*]^)〉 [19, 25], that is the average degree on layer *β* of a node with degree *k*^[*α*]^ on layer *α*. Again, 〈*k*^[*β*]^(*k*^[*α*]^)〉 will be an increasing function of *k*^[*α*]^ if nodes tend to have similar degrees on both layers (assortative inter-layer correlations), while 〈*k*^[*β*]^(*k*^[*α*]^)〉 will decrease with *k*^[*α*]^ if a hub on one layer will preferentially have small degree on the other layer, and vice-versa.

Finally, we measured the presence of mixed correlations through the function $\langle K_{nn}^{\left[\beta ,\alpha \right]}\left(k^{\left[\alpha \right]}\right)\rangle$, that is the average degree on layer *β* of the nearest neighbours on layer *β* of a node with degree *k*^[*α*]^ on layer *α* [26]. In analogy with the case of intra-layer correlations, the node term is $K_{nn,i}^{\left[\beta ,\alpha \right]}=\frac{\sum_{j\neq i}a_{ij}^{\left[\beta \right]}k_{j}^{\left[\beta \right]}}{k_{i}^{\left[\alpha \right]}}$. We remark here that there exists another possible definition of mixed correlations coefficient, which considers the nearest neighbours of a node on layer *α* rather then *β* (see Ref. [26] for details). The results for the alternative definition of mixed correlations are analogous to those observed for $\langle K_{nn}^{\left[\beta ,\alpha \right]}\left(k^{\left[\alpha \right]}\right)\rangle$ and are not shown in the text. In general, correlation functions might be affected by the degree sequence at each layer of the multiplex. In the simple scenario considered at first, however, we do not fit the parameter *m* from the data, to reduce as much as possible the complexity of the model. Instead, in order to still perform an accurate comparison between the synthetic multiplex networks constructed by our model and the real ones, in a second step we divided all the correlation functions by their (constant) value expected in the corresponding configuration model network. The correct normalisation for the intra-layer correlation function is $\frac{\langle {\left(k^{\left[\alpha \right]}\right)}^{2}\rangle }{\langle k^{\left[\alpha \right]}\rangle }$ [34], while for the inter-layer correlation function we have to divide 〈*k*^[*β*]^(*k*^[*α*]^)〉 by 〈*k*^[*β*]^〉. Finally, the mixed correlation function is correctly normalised by $\frac{\langle {\left(k^{\left[\beta \right]}\right)}^{2}\rangle }{\langle k^{\left[\alpha \right]}\rangle }$.
